# Oxidative Stress and Ultrastructural Analysis in Heart, Aorta, Skeletal Muscle and Lung of Rats Treated with N-Acetylcysteine or Rutin After Sprint Running

**DOI:** 10.3390/jfmk10020206

**Published:** 2025-06-02

**Authors:** Mădălina Moldovan, Mara Muntean, Sandra Andrea Schauer, Remus Moldovan, Daniela-Rodica Mitrea

**Affiliations:** 1 Department of Physiology, Iuliu Hatieganu University of Medicine and Pharmacy, Clinicilor Street, No. 1, 400006 Cluj-Napoca, Romania; moldovan.madalina@elearn.umfcluj.ro (M.M.);; 2 Department of Cell and Molecular Biology, Iuliu Hatieganu University of Medicine and Pharmacy, Pasteur Street, No. 6, 400349 Cluj-Napoca, Romania; muntean.mara@elearn.umfcluj.ro

**Keywords:** antioxidant, oxidative stress, running, intense, natural, artificial

## Abstract

**Background:** Sprinting, a high-intensity, short-duration exercise, induces oxidative stress. This causes molecular and ultrastructural alterations. Antioxidant supplementation may mitigate side effects of near or complete exhaustion. **Methods:** Twenty-eight healthy male adult rats received orally normal saline, carboxymethylcellulose (vehicle), artificial, N-acetylcysteine or a natural antioxidant, Rutin. Rats were subjected to treadmill sprinting at increasing speeds for 5 days/week. After 26 days, samples were collected to measure oxidative stress (malondialdehyde, MDA; the ratio of reduced-to-oxidized glutathione, GSH/GSSG), inflammation markers (enzymatic level of inducible nitric oxide synthase, iNOS; cytokine level of tumor necrosis factor alpha, TNFα) and for transmission electron microscopy (TEM) analysis. Results: Rutin attenuated MDA levels and increased antioxidant protection in all tissues, while NAC decreased the lipid peroxidation in all tissues except the lungs. NAC increased aortic inflammation, with higher TNF-α and iNOS. Sprinting caused intimal detachment in the heart and aorta. Rutin and NAC minimized endocardium alterations. Additionally, Rutin prevented myocardial disorganization. **Conclusions:** Rutin mitigated the oxidative stress damage of sprinting in the heart, aorta, skeletal muscle and lung. NAC protected against oxidative injury caused by sprinting in the heart, aorta and muscle but not the lung, and it induced aortic inflammation.

## 1. Introduction

Exercise induces oxidative stress, causing molecular and ultrastructural changes in the body [1,2,3]. Redox imbalance correlates with the intensity and duration of exercising [4]. Sprinting, a high-intensity, short-duration exercise, occurs in a mostly anaerobic state, pushing the body to fatigue [5,6]. The progression of inquiries is whether antioxidant supplementation is sufficient to counteract potential side effects associated with near or complete exhaustion [7,8,9].

Theories have been postulated for the mechanism through which physical activity generates reactive oxygen species (ROS) [10]. The bottom line is that the metabolic demand of physical exertion increases electron transport chain activity in the mitochondria, resulting in an augmentation of ROS [10]. In sprint training, due to its strenuous nature, the body shifts towards an anaerobic state and an increased adenosine triphosphate consumption [10]. This breakdown creates a chain of biproducts, adenosine diphosphate, adenosine monophosphate, adenosine, hypoxanthine, xanthine and uric acid [10]. In this molecular environment, extra-mitochondrial enzymes become active: hypoxanthine oxidase and NADPH oxidase—which generate ROS [10].

The body possesses an endogenous antioxidant system, including key enzymes such as superoxide dismutase, catalase and glutathione peroxidase, which are sufficient to maintain homeostasis under physiological conditions. Excessive ROS production through exercise may overwhelm this system, leading to oxidative stress and multi-organ damage [10]. Thus, the use of antioxidants among professional and recreational athletes is justified. Options aiming to enhance recovery range from synthetic to naturally sourced supplements [11].

Among the plant-derived antioxidants are polyphenols, which have a proven ability to suppress synthesis and scavenge ROS [12]. Through their meta-analysis of healthy patients, Somerville et al. determined that supplementation with polyphenols, especially quercetin, has athletic enhancing properties after one week of administration [13]. However, Bojarczuk et al. underlined the need for further studies as to assess the safety of polyphenols as dietary supplements, raising the possibility of their reclassification to medicinal substances [14]. This is because of potential side effects due to excessive antioxidant supplementation. Exercise requires an adaptive response by the body. The belief is that this adaptive mechanism is triggered by the actual production of ROS at the beginning of exertion. Thus, low levels of ROS are a requirement for physiological athletic activity [10].

N-acetylcysteine (NAC) is a commonly used synthetic antioxidant. Molecularly, it provides cysteine, the rate-limiting amino acid for synthesis of glutathione. Glutathione is an endogenous antioxidant tripeptide (glutamate, cysteine, glycine) that maintains redox balance by cycling between its reduced (GSH) and oxidized (GSSG) forms [15,16,17]. A previous systematic review by Fernandez-Lazaro et al. found that NAC demonstrated enhancements in exercise performance and antioxidant capacity [15]. Similarly, in their meta-analysis, Sadowski et al. concluded that NAC following exertion decreases muscle soreness, oxidative stress and inflammation [16]. The meta-analysis by Rhodes et al. found that NAC may be more effective when administered after near or complete exhaustion, such as following sprint training [17]. They also highlighted the need for further studies on its safety given the reported side effects [17].

In this study, we subjected healthy male rats to sprint running while administering Rutin or NAC. Rutin, a flavonol from the flavonoid subclass of dietary polyphenols, has shown antioxidant, cytoprotective, vasoprotective and cardioprotective benefits [18]. The effects were evaluated by measuring oxidative stress markers in samples from the lung, the gastrocnemius muscle, the thoracic aorta, the heart and aortic inflammatory markers. Additionally, transmission electron microscopy (TEM) was performed on all tissues.

## 2. Materials and Methods

### 2.1. Reagents

N-acetylcysteine (NAC), Rutin, carboxymethylcellulose (CMC) and the necessary chemicals for determination of antioxidant parameters were purchased from Merck (Darmstadt, Germany). Malondialdehyde (MDA) was determined with MDA colorimetric assay kit (TBA method) from Elabscience Biotechnology Inc. A (Houston, TX, USA), iNOS through Rat NOS2/iNOS (Nitric Oxide Synthase 2, Inducible) ELISA Kit and TNF-α using Rat TNF-α ELISA Kit from ORION Biologics S.R.L. (Cluj-Napoca, Romania).

### 2.2. Exercise Device and Software

For exerting the animals, we utilized a customized treadmill obtained from WATT Laboratory Solutions in Romania, Model LE8700TS, “Panlab/Harvard Apparatus”, equipped with SeDaCom v2.02 Soft software.

### 2.3. Experimental Design

The study respected the Directive 86/609/EEC and received authorization from the University Ethical Board and the Veterinary and Food Safety Direction (nr. 241/29.12.2020). Twenty-eight Wistar albino male rats, three months old, weighing 200 ± 20 g, were obtained from the Experimental Animal Facility of Iuliu Hatieganu University of Medicine and Pharmacy in Cluj-Napoca, Romania. The animals were hosted in cages under standard conditions (temperature of 21 ± 2 °C, relative humidity of 55 ± 5%), having ad libitum access to filtered tap water and standard chow. Prior to the experiment, rats were given one week of acclimatization.

The experiment consisted of 26 days of daily administration of substances by oral gavage. No animals were excluded from the study, as all subjects survived until the conclusion of the experiment. One of the authors (R.M.), an accredited animal researcher, was aware of the group allocations at the various stages of the experiment. The animals were randomly allocated into four groups (n = 7) as follows: NS (0.5 mL/day of 0.9% normal saline), CMC (0.5 mL/day of 1% carboxymethylcellulose vehicle solution), NAC (0.5 mL/day of 200 mg/day or 2.85 mg/kg/day of N-acetylcysteine) and Rutin (0.5 mL/day of 50 mg/kg/day of Rutin). The pharmacokinetic properties of NAC and Rutin are different, thus the need for different suspensions. Rutin was suspended in CMC because of its lipophilia, while NAC was delivered via normal saline solution because it is highly hydrophilic. Following administration, animals were allowed one hour of rest before undertaking the allotted physical exercise. The horizontal treadmill, with a negative reward system built-in, ensured a constant exercise rate. Thus, once the rat stopped moving and reached the edge of the treadmill, it received a non-harmful electric impulse of 1 mA. To evade this stimulus, the animal persists in running until a predetermined 5-min timeframe elapses. Increasing speeds within the sprinting range were applied during the 4 weeks (Table 1).

On the 26th day of the experiment, blood was collected from the retroorbital plexus immediately after the animal stopped running. Then, animals were sacrificed using an overdose of ketamine 10% (5 mg/100 gbw) and xylazine hydrochloride 2% (100 mg/100 gbw). Subsequently, samples of lung parenchyma, gastrocnemius muscle, thoracic aorta and heart tissue were collected.

### 2.4. Oxidative Stress and Inflammation Analysis

Oxidative stress was investigated through MDA [19], GSH [20], GSSG [21] and GSH/GSSG ratio. MDA was measured using the fluorimetric method with 2-thiobarbituric acid as described by Conti et al. [19]. Samples were mixed with a 10 mM solution of 2-thiobarbituric acid in 75 mM K_2_HPO_4_, pH 3, and heated in a boiling water bath for 1 h. After cooling, the reaction products were extracted into n-butanol. MDA was determined spectrofluorimetrically in the organic phase using a synchronous technique with excitation at 534 nm and emission at 548 nm. MDA values were expressed as nmol/mg protein. GSH levels in plasma were determined using a sensitive fluorometric assay adapted from tissue GSH methods to accommodate the low micromolar concentrations present in plasma. Plasma proteins were precipitated with cold 10% trichloroacetic acid (TCA), followed by centrifugation to obtain a clear supernatant. The supernatant was then reacted with o-phthalaldehyde in a phosphate-EDTA buffer (pH 8.0), and fluorescence was measured at an excitation wavelength of 350 nm and emission at 420 nm. GSSG levels in whole blood were determined using the following fluorometric method. After protein precipitation with 10% m-phosphoric acid and centrifugation, 250 µL of the supernatant was incubated with 0.1 mL of 40 nmol/L N-ethylmaleimide for 30 min to block free GSH. Then, 0.65 mL of 0.1 N NaOH was added, followed by the addition of o-phthalaldehyde. Fluorescence was measured at an excitation wavelength of 350 nm and an emission wavelength of 420 nm. Unlike in the GSH assay, 0.1 M NaOH was used instead of phosphate buffer for fluorescence development. Additionally, the assay of TNF-α, iNOS, was performed in aorta using spectrometer-based ELISA readers (37 °C, 450 nm absorbance) with Magellan^TM^ data Analysis software V6.3, Tecan Trading AG, Männedorf, Switzerland.

### 2.5. Transmission Electron Microscopy Analysis

Samples from lung, muscle, aorta and heart were initially fixed in a 2.7% glutaraldehyde solution in 0.1 M phosphate buffer. Then, the samples underwent 4 washes in the same buffer. For postfixation, a 1.5% osmium tetroxide (OsO4) dissolved in 0.15 M phosphate buffer was used. The samples were then dehydrated using a series of acetone solutions with increasing concentrations (ranging from 30% to 100%), followed by infiltration and embedding in EMbed 812. Ultrathin sections measuring 70–80 nm in thickness were obtained using a Diatome A382 diamond knife (Diatome, Hatfield, PA, USA) on a Bromma 8800 ULTRATOMEIII (LKB, Stockholm, Sweden). These sections were subsequently collected onto 300 mesh copper grids, contrasted with uranyl acetate and lead citrate and examined at 80 kV using a JEOL JEM-100CX II transmission electron microscope (JEOL, Tokyo, Japan). Images were captured using a MegaView G3 camera fitted with Radius 2.1 software, both supplied by Emsis (Münster, Germany).

### 2.6. Statistical Analysis

Oxidative stress and inflammation parameters were analyzed by one-way ANOVA followed by the Tukey Post test. The distances covered were evaluated using two-way ANOVA followed by the Bonferroni Post tests. GraphPad Prism version 5.03 for Windows, GraphPad Software (San Diego, CA, USA) was employed. The significance threshold was established at *p* < 0.05.

## 3. Results

### 3.1. Treadmill Running

**Figure 1:**
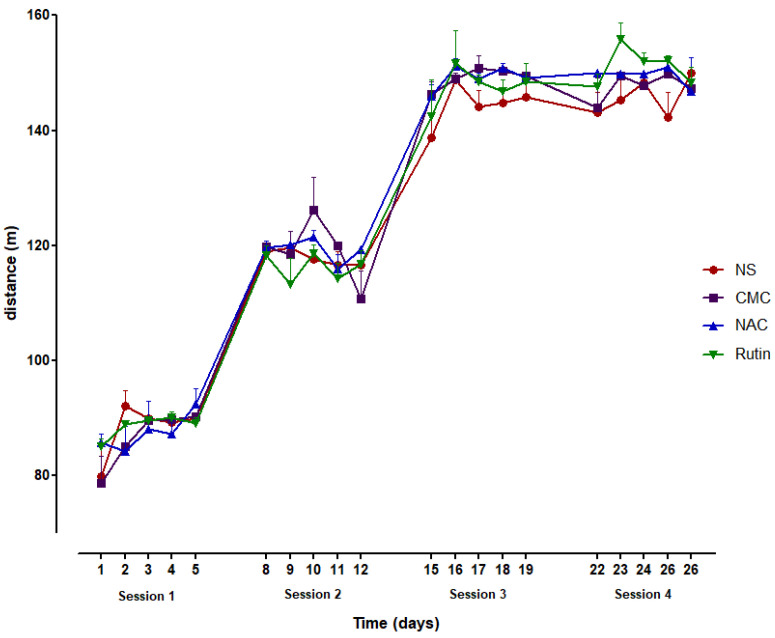
The distance covered during the 4 running sessions (5 days/session) in groups of normal saline (NS), carboxymethylcellulose (CMC), N-acetylcysteine (NAC) and Rutin.

Administration of NAC or Rutin resulted in a non-significant increase in the distance travelled during the entire running period. More noticeable outcomes were observed in the latter phase of the experiment (Figure 1).

### 3.2. Physical Effort Effects on the Heart

#### 3.2.1. Oxidative Stress in Heart

**Figure 2:**
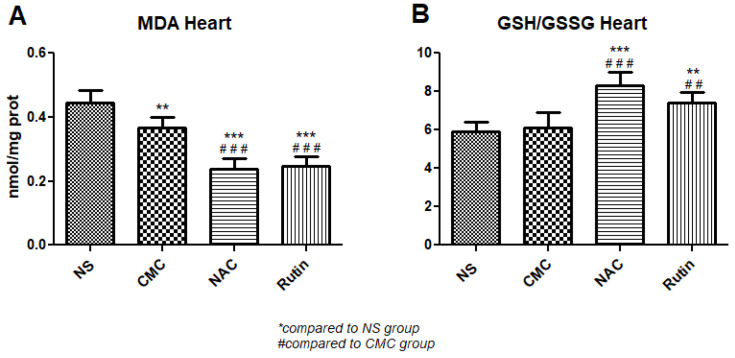
Oxidative stress markers in the heart: malondialdehyde (MDA) (**A**) and the reduced-to-oxidized glutathione ratio (GSH/GSSG) (**B**) in the groups normal saline (NS), carboxymethylcellulose vehicle (CMC), N-acetylcysteine (NAC) and Rutin. Rutin and NAC significantly decreased MDA levels and increased the GSH/GSSG ratio in the heart following intense physical exertion (** *p* < 0.01, *** *p* < 0.001 compared to NS; ## *p* < 0.01, ### *p* < 0.001 compared to CMC).

In heart, administration of NAC decreased MDA (*p* < 0.001) and increased the GSH/GSSG ratio (*p* < 0.001), compared to the NS group. Similarly, Rutin lowered MDA levels (*p* < 0.001) and increased the GSH/GSSH ratio (*p* < 0.01), compared to the CMC group (Figure 2).

#### 3.2.2. Heart Transmission Electron Microscopy

**Figure 3:**
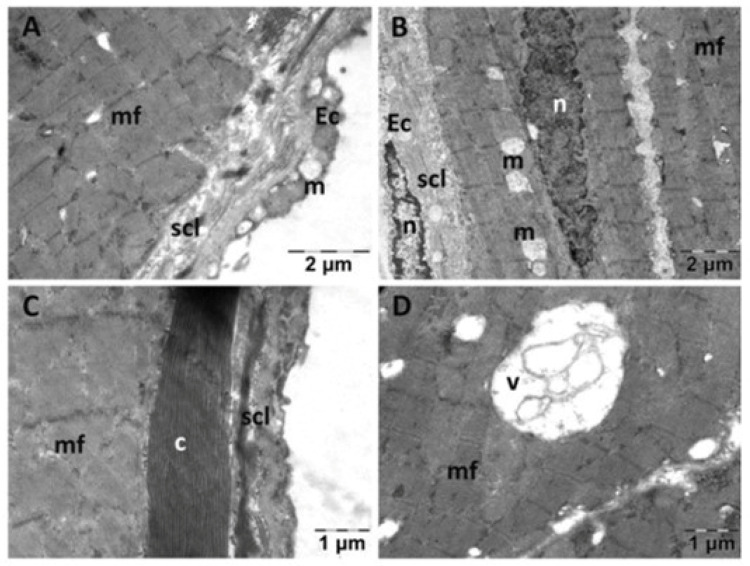
Transmission electron microscopy of the heart in the normal saline (NS) group following intense physical exertion. Endocardial endothelium shows damaged mitochondria (**A**,**B**,**D**) with increased subendothelial distance and thickness (**B**,**C**), while myocardial myofibrils appear normal (c: collagen fibers; Ec: endothelial cell; m: mitochondria; mf: muscle fibers; n: nucleus; scl: subendothelial connective layer; v: vacuolization).

In the NS group (Figure 3), the endocardium presented focal alterations. Endothelial cells had damaged mitochondria (Figure 3A) and were distanced from the underlying structures (Figure 3B). There were regions in which the subendothelial layer was rarefied (Figure 3B) or contained large amounts of thicker, parallel collagen fibers (Figure 3C). In the myocardium, the nucleus and the myofibrils displayed a normal structure. However, there were regions in which the smooth endoplasmic reticulum (SER) was dilated, and the mitochondria appeared swollen, with electron lucent matrix and disorganized cristae (Figure 3A,B,D). Extensive electron lucent regions were also identified in the myocardial tissue (Figure 3B,D). All these above-described changes were limited to areas of the myocardium, the rest of the tissue having a normal appearance.

**Figure 4:**
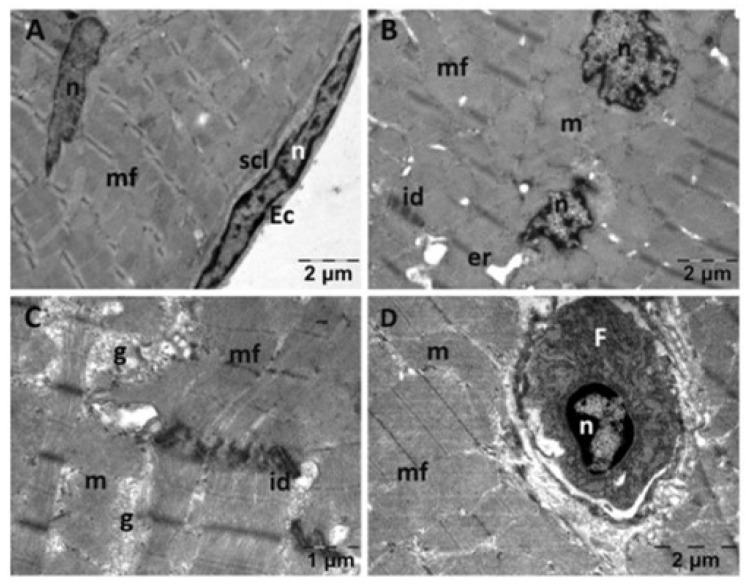
Transmission electron microscopy of the heart in the carboxymethylcellulose (CMC) group following intense physical exertion. The endocardium had a normal endothelium with reduced subendothelial thickness (**A**), while the myocardium displayed disorganized myofibrils (**B**,**C**). Fibroblasts were observed between heart muscle cells (**D**). (Ec: endothelial cell; er: endoplasmic reticulum; F: fibroblast; g: glycogen; id: intercalated disks; m: mitochondria; mf: muscle fibers; n: nucleus; scl: subendothelial connective layer).

In the CMC group (Figure 4), the endothelial cells appeared normal. The subendothelial layer had a reduced thickness (Figure 4A). Myofibrils appeared disorganized and discontinuous in certain areas (Figure 4C). Rare electron lucent regions were noted among the myofibrils (Figure 4B), and the cells appeared separated from each other at the level of the intercalary discs (Figure 4C). The mitochondria of muscle cells in this group appeared unaltered, and there were some enlargements of the SER, but they were fewer and smaller in diameter in comparison to group NS. Between the cardiac muscle cells, fibroblasts were observed, even though there did not seem to be an increase in the amount of collagen in the tissue (Figure 4D).

**Figure 5:**
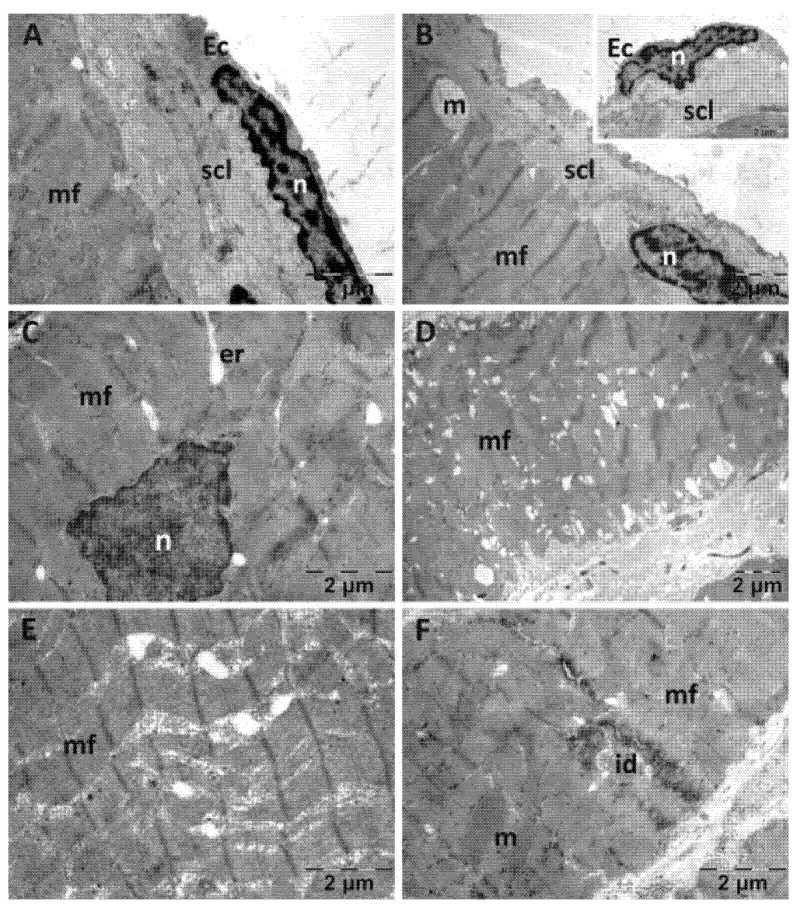
Transmission electron microscopy of the heart in the N-acetylcysteine (NAC) group following intense physical exertion. The endocardium has a normal endothelium and subendothelium (**A**), while the myocardium shows swollen mitochondria (**B**), enlarged smooth endoplasmic reticulum (**C**), minor disorganization of myofibrils (**D**,**E**) and large spaces between the dense plaques of the intercalated disks (**F**) (Ec: endothelial cell; er: endoplasmic reticulum; id: intercalated disks; m: mitochondria; mf: muscle fibers; n: nucleus; scl: subendothelial connective layer).

The endocardium from the NAC group displayed a normal structure (Figure 5). There were some alterations in the structure of the myocardium. Few mitochondria were swollen and had disorganized cristae and a less dense matrix (Figure 5B). The enlargements of the SER were larger and more numerous (Figure 5C), mixed with electron lucent spaces among myofibrils (Figure 5D). There were areas of an almost normal structure. Certain myofibrils appeared disorganized (Figure 5D,E), and larger spaces were seen between dense plaques of the intercalated disks (Figure 5F).

**Figure 6:**
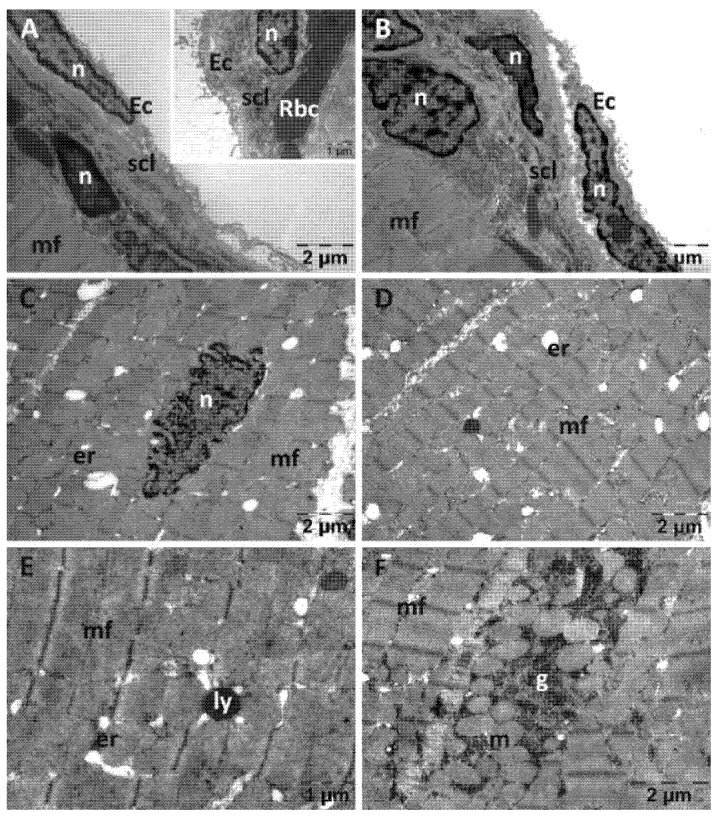
Transmission electron microscopy of the heart in the Rutin group following intense physical exertion. The endocardium shows a slight increase in subendothelial distance and increased subendothelial thickness (**A**,**B**), while the myocardium exhibits minor changes (**C**–**E**). In some areas, glycogen granules accumulated between myofibrils (**F**). (Ec: endothelial cell; er: endoplasmic reticulum; g: glycogen; ly: lysosome; m: mitochondria; mf: muscle fibers; n: nucleus; scl: subendothelial connective layer; Rbc: red blood cell).

In the Rutin group (Figure 6), the endothelial cells were slightly detached from the subendothelial layer that contained a larger amount of collagen fibers (Figure 6A,B). In a few places, fibroblasts (Figure 6A) or erythrocytes were observed in the subendothelial layer (inset in Figure 6A). The nuclei (Figure 6C), myofibrils and mitochondria of the smooth muscle cells from the myocardium (Figure 6D) all displayed a normal structure. In some areas, a large quantity of glycogen granules was observed between the myofibrils (Figure 6F).

### 3.3. Physical Effort Effects on the Aorta

#### 3.3.1. Oxidative Stress and Inflammation in the Aorta

**Figure 7:**
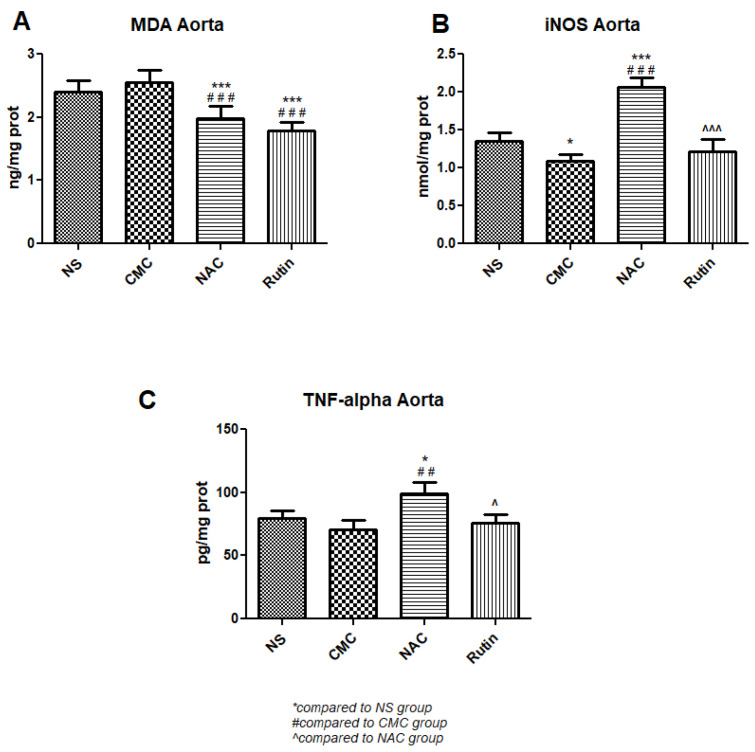
Oxidative stress and inflammation markers in the aorta: malondialdehyde (MDA) (**A**), inducible nitric oxide synthase (iNOS) (**B**) and tumor necrosis factor alpha (TNFα) (**C**) in the groups normal saline (NS), carboxymethylcellulose vehicle (CMC), N-acetylcysteine (NAC) and Rutin. Rutin and NAC decreased MDA levels in the aorta following intense physical exertion. Rutin had no protective effects against the aortic inflammation caused by sprinting, while NAC increased both iNOS and TNFα levels (* *p* < 0.05, *** *p* < 0.001 compared to NS; ## *p* < 0.01, ### *p* < 0.001 compared to CMC; ^ *p* < 0.05, ^^^ *p* < 0.001 compared to NAC).

There was a significant MDA reduction in treated exercised rats, NAC (*p* < 0.001) and Rutin (*p* < 0.001), when compared to untreated exercised rats. While Rutin did not modify inflammation markers, NAC significantly increased both iNOS (*p* < 0.001) and TNF-α (*p* < 0.01) compared to untreated exercised rats (Figure 7).

#### 3.3.2. Aorta Transmission Electron Microscopy

**Figure 8:**
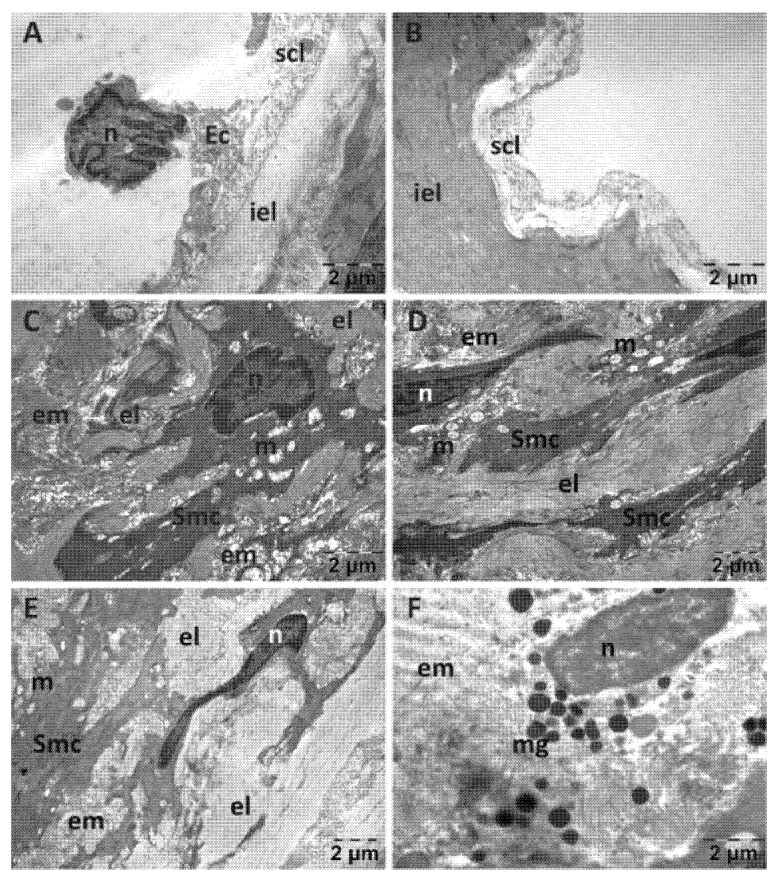
Transmission electron microscopy of the aorta in the normal saline (NS) group following intense physical exertion. The endothelial cells were prominent in the lumen (**A**) and in several places detached from the subendothelial layer (**B**). The subendothelial layer was thick and disorganized and smooth muscle cells appeared with abnormal structure (**C–E**). Mast cells with granules were also present (**F**). (Ec: endothelial cell; el: elastic lamina; em: extracellular matrix; iel: internal elastic lamina; m: mitochondria; mg: mast cell granules; n: nucleus; scl: subendothelial connective layer; Smc: smooth muscle cell).

For the NS group, endothelial cells were often prominent in the lumen of the aorta (Figure 8A), distanced or even detached from the subendothelial layer and absent in places (Figure 8B). The subendothelial layer was substantially reduced in thickness and often disorganized, in some areas to such an extent that the inner elastic lamina was denuded. The changes in the structure of the media were most evident in the smooth muscle cells. They displayed aberrant shapes, polymorphous nuclei with enlarged perinuclear space and ballooned mitochondria with a rarefied matrix (Figure 8C–E). Mast cell with numerous granules were also identified (Figure 8F).

**Figure 9:**
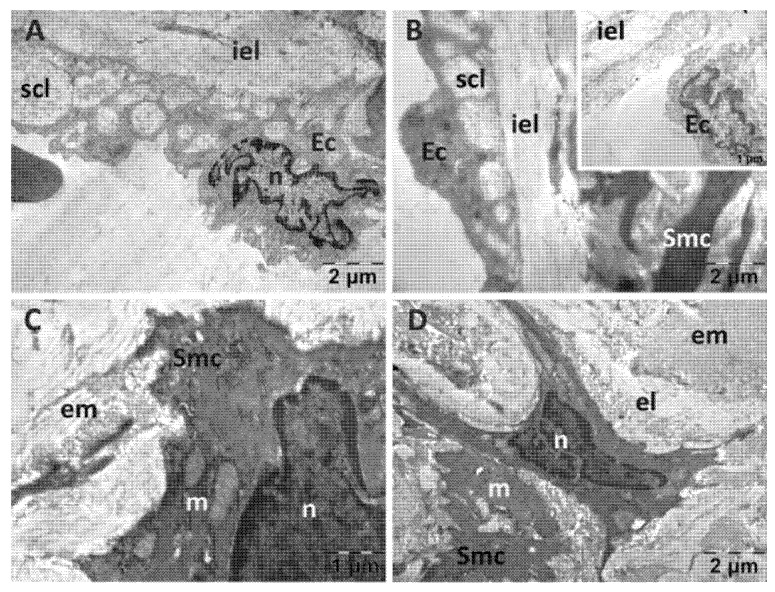
Transmission electron microscopy of the aorta in the carboxymethylcellulose (CMC) group following intense physical exertion. The endothelial cells were detached from the subendothelial layer (**A**,**B**). The subendothelial layer was disorganized ((**B**), insert). The smooth muscle cells had altered mitochondria (**C**,**D**) and intercellular matrix presented areas of a lower density (**D**). (Ec: endothelial cell; el: elastic lamina; em: extracellular matrix; iel: internal elastic lamina; m: mitochondria; n: nucleus; scl: subendothelial connective layer; Smc: smooth muscle cell).

In the vehicle group (Figure 9), the endothelial cells were distanced from the subendothelial layer and emitted prolongations towards the inner elastic lamina (Figure 9A,B). In the cytoplasm they presented Weibel-Palade bodies, showing a tendency of the endothelium to recover. There were areas with a disorganized subendothelial layer and endothelial cells that were separated from the underlying structures (insert in Figure 9B). The smooth muscle cells displayed fewer affected mitochondria, and the perinuclear space appeared normal in most cells (Figure 9C,D). The intercellular matrix contained large amounts of collagen fibers and presented areas of a lower density (Figure 9D).

**Figure 10:**
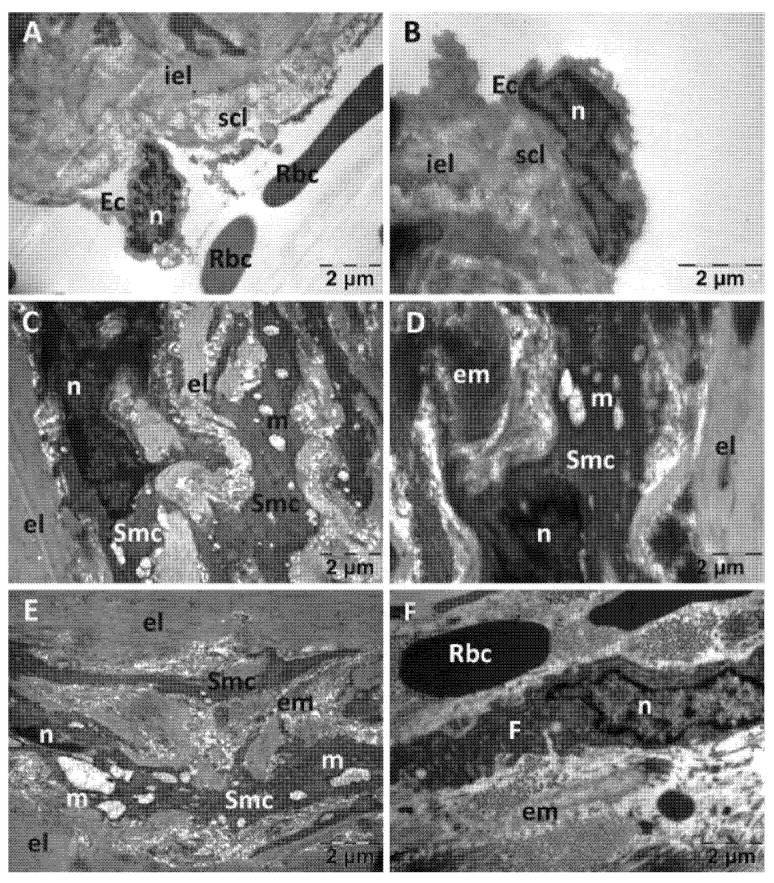
Transmission electron microscopy of the aorta in the N-acetylcysteine (NAC) group following intense physical exertion. The endothelium wasdetached; the subendothelium was disorganized (**A**,**B**), the smooth muscle cell had altered mitochondria (**C**) and a disorganized aspect (**D**,**E**). The extracellular matrix had an abnormal aspect (**F**). (Ec: endothelial cell; el: elastic lamina; em: extracellular matrix; F: fibroblast; iel: internal elastic lamina; m: mitochondria; n: nucleus; Rbc: red blood cell; scl: subendothelial connective layer; Smc: smooth muscle cell.).

The NAC group (Figure 10) showed detachment of the endothelial cells from the subjacent structures, a reduced thickness of their prolongations and a disorganization of the subendothelial layer and a variable thickness of the inner elastic lamina (Figure 10A,B). The smooth muscle cells displayed swollen mitochondria with disrupted cristae and an electron-lucent matrix (Figure 10C–E). A large amount of collagen was observed between the smooth muscle cells (Figure 10D,E) as well as occasional fibroblasts (Figure 10F), but the inflammatory cells were absent.

**Figure 11:**
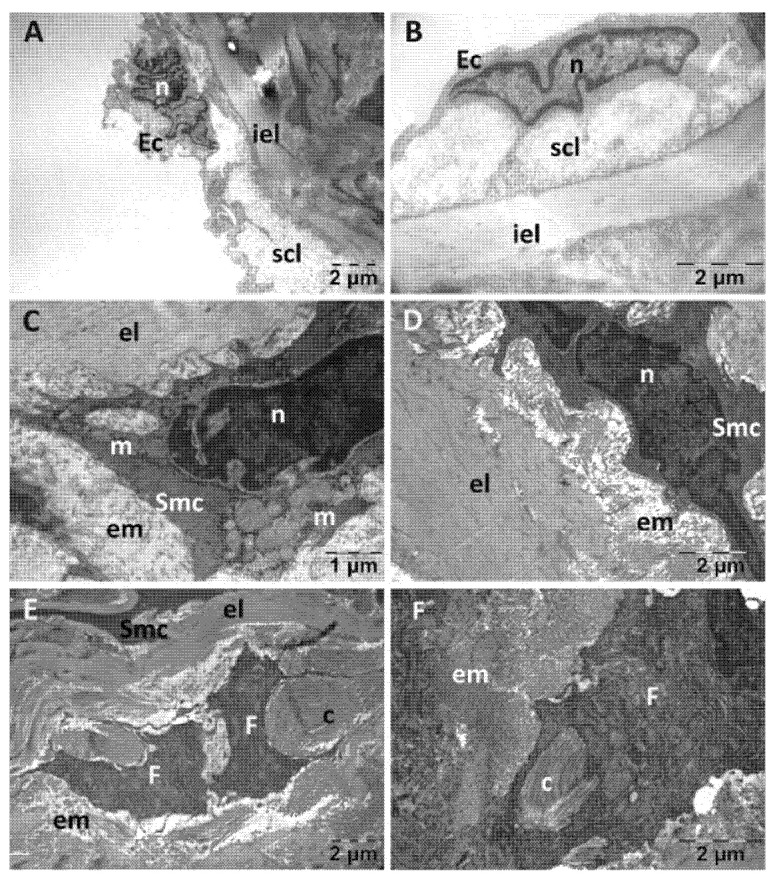
Transmission electron microscopy of the aorta in the Rutin group following intense physical exertion. The endothelium was mostly normal (**A**,**B**); but the subendothelium showed increased distance (**A**). The smooth muscle cells had a normal structure, with only some places where mitochondria were swollen (**C**,**D**). Fibroblasts and collagen fibers were accumulated between the elastic laminae (**E**,**F**). (c: collagen fibers; Ec: endothelial cell; el: elastic lamina; em: extracellular matrix; F: fibroblast; iel: internal elastic lamina; m: mitochondria; n: nucleus; scl: subendothelial connective layer; Smc: smooth muscle cell).

The endothelial cells in the Rutin group (Figure 11) were distanced from the subendothelial layer, were prominent in the lumen of the aorta and emitted prolongations towards the internal elastic lamina (Figure 11A,B). The thickness of the subendothelial layer was variable, and there were also fewer collagen fibers (Figure 11A). The structure of the smooth muscle cells was close to normal, with the perinuclear space and most of the mitochondria displaying a normal structure; however, a few ballooned mitochondria were observed (Figure 11C,D). Between the elastic laminae, numerous fibroblasts were present as well as large amounts of collagen fibers (Figure 11E,F).

### 3.4. Physical Effort Effects in the Lung

#### 3.4.1. Oxidative Stress in the Lung

**Figure 12:**
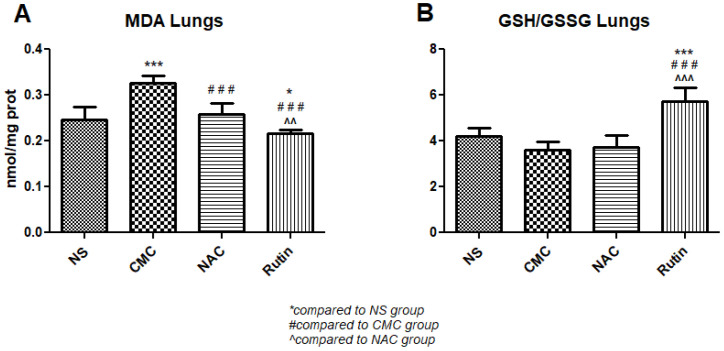
Oxidative stress markers in the lung: malondialdehyde (MDA) (**A**) and the reduced-to-oxidized glutathione ratio (GSH/GSSG) (**B**) in the groups normal saline (NS), carboxymethylcellulose vehicle (CMC), N-acetylcysteine (NAC) and Rutin. Rutin decreased MDA levels and increased the GSH/GSSG ratio, while NAC had no effect on these markers in the lung following intense physical exertion (* *p* < 0.05, *** *p* < 0.001 compared to NS; ### *p* < 0.001 compared to CMC; ^^ *p* < 0.01, ^^^ *p* < 0.001 compared to NAC).

In the lung, NAC had no effect on oxidative stress. When compared to the vehicle, Rutin showed lower levels of MDA (*p* < 0.01) and an increase in the GSH/GSSH ratio (*p* < 0.001). Compared to NAC, Rutin decreased MDA (*p* < 0.001) and increased the GSH/GSSH ratio (*p* < 0.001) (Figure 12).

#### 3.4.2. Lung Transmission Electron Microscopy

**Figure 13:**
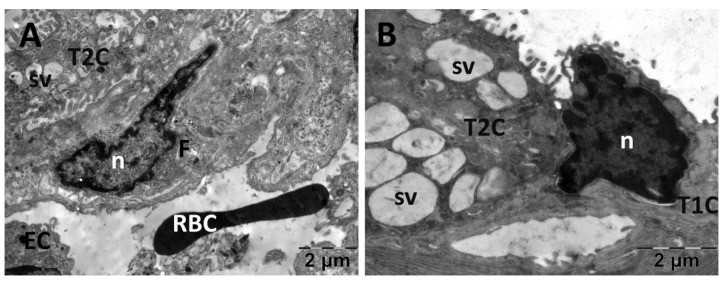
Transmission electron microscopy of the lung in the normal saline (NS) group following intense physical exertion The lung tissue presented an aspect close to normal (**A**,**B**) with several fibroblasts (**A**) and vacuoles (**B**). (F: fibroblast; n: nucleus; RBC: red blood cell; sv: surfactant vesicles; T1C: alveolar cell type 1; T2C: alveolar cell type 2).

**Figure 14:**
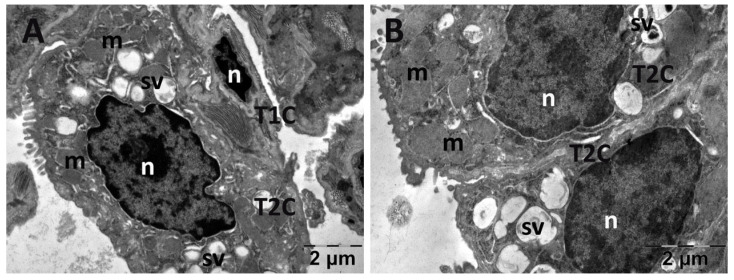
Transmission electron microscopy of the lung in the carboxymethylcellulose (CMC) group following intense physical exertion. The lungs had an almost normal aspect, but the surfactant vesicles were not so abundant (**A**,**B**) (m: mitochondria; n: nucleus; sv: surfactant vesicles; T1C: alveolar cell type 1; T2C: alveolar cell type 2).

**Figure 15:**
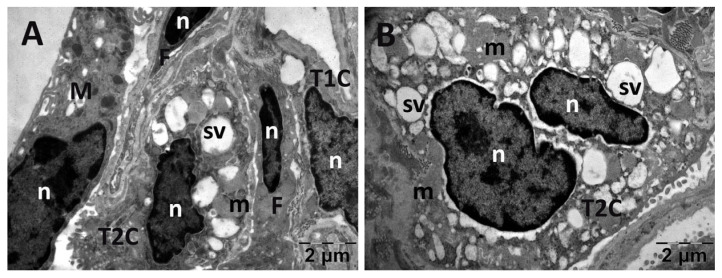
Transmission electron microscopy of the lung in the N-acetylcysteine (NAC) group following intense physical exertion. The lungs had an increased number of vacuoles (**A**,**B**) (F: fibroblast; M: macrophage; m: mitochondria; n: nucleus; sv: surfactant vesicles; T1C: alveolar cell type 1; T2C: alveolar cell type 2).

**Figure 16:**
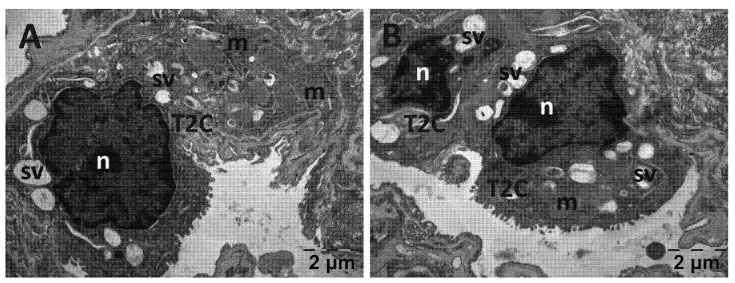
Transmission electron microscopy of the lung in the Rutin group following intense physical exertion. The aspect of the lungs was almost normal, with several vacuoles (**A**,**B**). (m: mitochondria; n: nucleus; sv: surfactant vesicles; T2C: alveolar cell type 2).

For the lungs, across all groups, alveolar type II cells exhibited an absence of the typical vesicular surfactant appearance (Figure 13, Figure 14, Figure 15 and Figure 16). Furthermore, in the NAC group, a cell was observed exhibiting vacuolization and an enlarged perinuclear space (Figure 15). In the NS group, the lung tissue was normal except for occasional fibroblasts (Figure 13).

### 3.5. Physical Effort Effects on the Gastrocnemius Muscle

#### 3.5.1. Oxidative Stress in the Gastrocnemius Muscle

**Figure 17:**
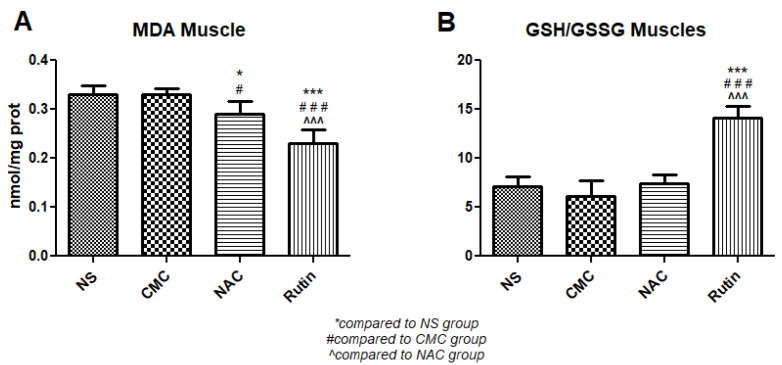
Oxidative stress markers in the gastrocnemius muscle: malondialdehyde (MDA) (**A**) and the reduced-to-oxidized glutathione ratio (GSH/GSSG) (**B**) in the groups normal saline (NS), carboxymethylcellulose vehicle (CMC), N-acetylcysteine (NAC) and Rutin. Rutin decreased MDA levels and increased the GSH/GSSG ratio, while NAC only decreased MDA (* *p* < 0.05, *** *p* < 0.001 compared to NS; # *p* < 0.05, ### *p* < 0.001 compared to CMC; ^^^ *p* < 0.001 compared to NAC).

The administration of NAC to rats after physical effort resulted in lower MDA in the gastrocnemius muscle (*p* < 0.05) compared to the vehicle. Compared to the vehicle, Rutin reduced MDA levels (*p* < 0.001) and increased the GSH/GSSH ratio (*p* < 0.001). Compared to NAC, Rutin decreased MDA (*p* < 0.001) and increased the GSH/GSSH ratio (*p* < 0.001) (Figure 17).

#### 3.5.2. Muscle Transmission Electron Microscopy

**Figure 18:**
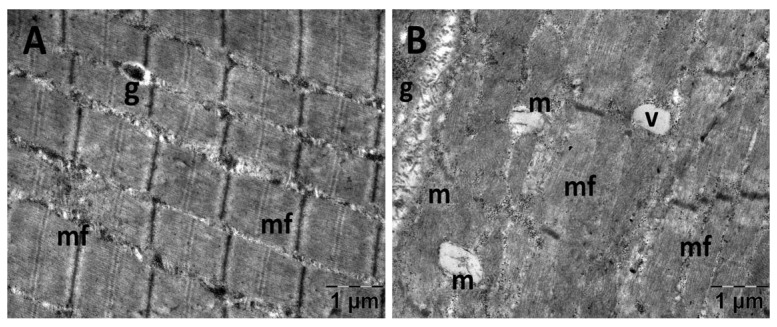
Transmission electron microscopy of the gastrocnemius muscle in the normal saline (NS) group following intense physical exertion, displaying a mostly normal appearance (**A**,**B**) (g: glycogen granules; m: mitochondria; mf: muscle fibers; v: vacuolization).

**Figure 19:**
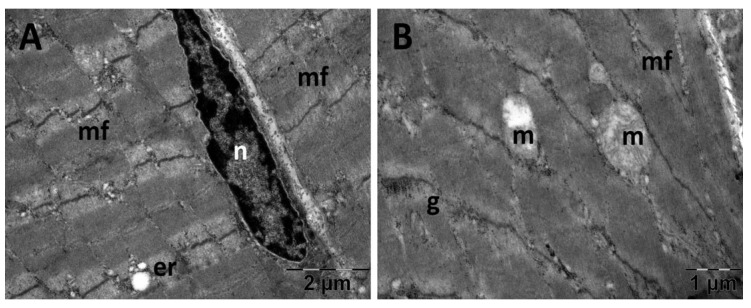
Transmission electron microscopy of the gastrocnemius muscle in the carboxymethylcellulose (CMC) group following intense physical exertion. The skeletal muscle appeared close to normal (**A**) but with several swollen mitochondria (**B**) (er: endoplasmic reticulum; g: glycogen granules; m: mitochondria; mf: muscle fibers; n: nucleus).

**Figure 20:**
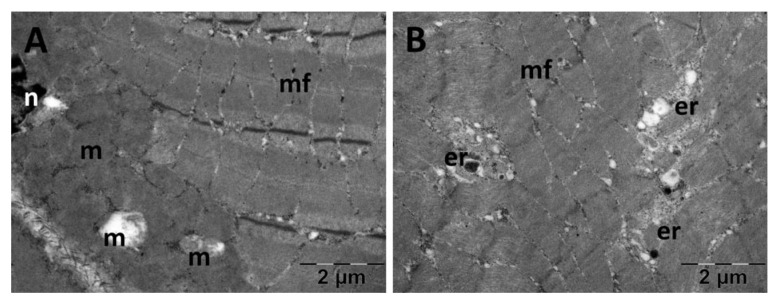
Transmission electron microscopy of the gastrocnemius muscle in the N-acetylcysteine (NAC) group following intense physical exertion. The skeletal muscle showed enlarged mitochondria (**A**,**B**). (er: endoplasmic reticulum; m: mitochondria; mf: muscle fibers; n: nucleus).

**Figure 21:**
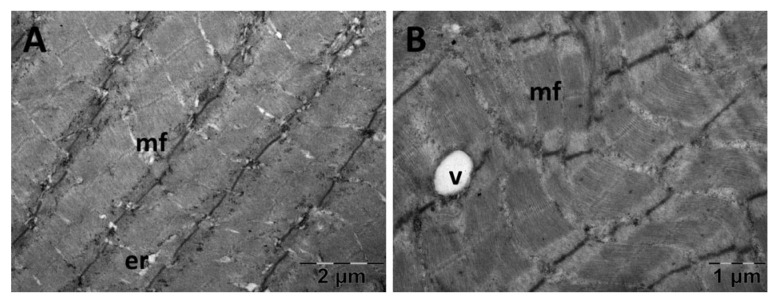
Transmission electron microscopy of the gastrocnemius muscle in the Rutin group following intense physical exertion. The skeletal muscle had a normal aspect (**A**) with rare vacuoles (**B**) displaying the protective effects of Rutin administration. (er: endoplasmic reticulum; mf: muscle fibers; v: vacuolization).

For the gastrocnemius muscle, a zonal pattern of vacuolization is observable in both the NS (Figure 18) and Rutin groups (Figure 21). The occurrences of enlarged vacuolated mitochondria are evident (Figure 18, Figure 19, Figure 20 and Figure 21). Rutin exhibited a protective effect (Figure 21). In the NS group, the muscle tissue had a normal appearance (Figure 18).

## 4. Discussion

This study explored the natural (Rutin) and artificial (NAC) antioxidants in healthy male rats subjected to treadmill sprinting to the point of oxidative stress imbalance. Samples from the heart, aorta, skeletal muscle and lung were analyzed molecularly and ultrastructurally using TEM.

The administration of NAC or Rutin produced a slight increase in the total distance covered during the running sessions, with improvements observed in the later stages of the experiment. These may be associated with cardio-respiratory conditioning, although additional evidence is needed.

The redox status reflects the balance between ROS production and antioxidant defense. Glutathione is an endogenous intracellular antioxidant that is converted from a reduced (GSH) to an oxidized state (GSSG) as it neutralizes ROS. The oxidation of glutathione is catalyzed by glutathione peroxidase (GPx) and the restoration by glutathione reductase [20,21]. Thus, a high GSH/GSSG ratio indicates a protective, reduced state (with higher GSH), while a low ratio signals oxidative stress (with higher GSSG) [20,21]. When the endogenous antioxidant defense is overwhelmed, ROS causes lipid peroxidation, marked by an increase in MDA. The state of glutathione indicates the body’s initial response to redox imbalance, which then progresses to cellular damage marked by higher MDA levels. Rutin and NAC have different pharmacological mechanisms of action. NAC serves as a source of cysteine, one of the key precursors of GSH. Conversely, Rutin acts through multiple mechanisms, as it increases GPx activity, replenishes GSH and actively scavenges ROS [22].

Both NAC and Rutin reduced cardiac oxidative stress by lowering MDA and increasing the GSH/GSSG ratio. However, exercise caused a disorganization of cardiac myofibrils, noticeable upon TEM, which was not restored by NAC. These results are in accordance with the findings by Marian et al., who stated that NAC supplementation in hypertrophic cardiomyopathy did not lower fibrosis significantly [23]. Arica et al. found similar results in a rat model of doxorubicin cardiac toxicity, where NAC failed to prevent myofibril disorganization [24]. Rutin preserved normal myofibril structure in most instances, with only minor changes visible [25,26,27]. This is in accordance with its proven cardioprotective properties, as it opposes myocyte apoptosis [28,29] and preserves myocardium structure [30,31]. Notably, CMC, the vehicle used to deliver Rutin, caused marked myocardial disorganization. Previously, it has been noted that CMC increases the risk of cardiovascular disease. However, there is still debate regarding the pathophysiology involved. It is likely to involve microbiota dysbiosis and intestinal inflammation [32].

Sprinting caused severe alterations in the intima layer of the aorta across all groups. The endothelium was distanced or completely detached, with areas of denudation. No treatment could oppose these effects despite both NAC and Rutin being aortic antioxidants. However, despite its antioxidant properties, NAC increased the pro-inflammatory necrosis factor-alpha (TNF-α). Similar results were found by Gunturk et al. in a rat model of cisplatin cardiotoxicity [33]. They found that NAC increased nuclear factor kappa B (NF-kB), which is canonically activated by high levels of TNF-α [33], a mechanism that could explain our findings. The NF-κB pathway, upon activation, translocates to the nucleus, where it triggers the expression of genes involved in inflammation, apoptosis and immune responses [33]. In the context of inflammation, NAC increased the expression of iNOS, leading to elevated NO. While NO plays important physiological roles, excessive amounts generated by iNOS can react with superoxide anions to form peroxynitrite (ONOO^−^), a highly reactive and damaging oxidant. Peroxynitrite contributes to oxidative and nitrosative stress, promoting lipid peroxidation, protein nitration and mitochondrial dysfunction [34].

Rutin was the sole lung antioxidant. Similar results were found by Tosun et al., where Rutin mitigated the lung oxidative stress in a desflurane pulmonary injury rat model [35]. Bai et al. showed that in a rat model of bleomycin pulmonary fibrosis, Rutin protected the lungs by lowering MDA and increasing GSH [36].

In the gastrocnemius muscle, NAC decreased MDA. According to a systematic review by Fernández-Lázaro et al., individuals who received NAC had enhanced exercise performance, antioxidant capacity and GSH levels [15]. Similarly, Aksu et al. found that in a rat model of gastrocnemius ischemia/reperfusion, NAC reduced MDA [37]. Rutin was a superior muscle antioxidant, as confirmed by TEM, which showed preserved muscle structure.

The limitations of this study warrant further investigation. Future research directions could explore the pharmacological differences between NAC and Rutin.

## 5. Conclusions

Our study demonstrated that progressive treadmill sprint running caused multi-organ alterations in a healthy male rat model. These modifications were recorded in the heart, thoracic aorta, lungs and gastrocnemius muscle through oxidative stress, inflammation and transmission electron microscopy examination. Exercise without supplementation showed higher levels of tissue damage, most notably in the aorta, where areas of endothelium detachment were noted. Supplementation with Rutin throughout the exercise period had the best overall effects, acting as an antioxidant in all investigated tissues. The administration of NAC did not have any beneficial effects in the lungs and caused a pro-inflammatory state in the aorta.

## Figures and Tables

**Table 1:** Treadmill running protocol: duration of exercise was 5 min/round/animal 5 days/week; belt velocity was increased weekly for three weeks (30 cm/s, 40 cm/s, 50 cm/s), the 4th week being kept at a maximum (50 cm/s).

Week	Belt Velocity (cm/s)	Duration (min)	Frequency (days/week)
1	30	5	5
2	40	5	5
3	50	5	5
4	50	5	5

## Data Availability

Data are contained within the article.
